# Fixations on objects in natural scenes: dissociating importance from salience

**DOI:** 10.3389/fpsyg.2013.00455

**Published:** 2013-07-19

**Authors:** Bernard M. 't Hart, Hannah C. E. F. Schmidt, Christine Roth, Wolfgang Einhäuser

**Affiliations:** ^1^Neurophysics, Philipps-University MarburgMarburg, Germany; ^2^Physiological Genomics, Ludwig Maximilian UniversityMunich, Germany; ^3^Neurology, Philipps-University MarburgMarburg, Germany; ^4^Center for Interdisciplinary Research (ZiF)Bielefeld, Germany

**Keywords:** attention, eye movements, natural scenes, luminance contrast, salience, objects

## Abstract

The relation of selective attention to understanding of natural scenes has been subject to intense behavioral research and computational modeling, and gaze is often used as a proxy for such attention. The probability of an image region to be fixated typically correlates with its contrast. However, this relation does not imply a causal role of contrast. Rather, contrast may relate to an object's “importance” for a scene, which in turn drives attention. Here we operationalize importance by the probability that an observer names the object as characteristic for a scene. We modify luminance contrast of either a frequently named (“common”/“important”) or a rarely named (“rare”/“unimportant”) object, track the observers' eye movements during scene viewing and ask them to provide keywords describing the scene immediately after. When no object is modified relative to the background, important objects draw more fixations than unimportant ones. Increases of contrast make an object more likely to be fixated, irrespective of whether it was important for the original scene, while decreases in contrast have little effect on fixations. Any contrast modification makes originally unimportant objects more important for the scene. Finally, important objects are fixated more centrally than unimportant objects, irrespective of contrast. Our data suggest a dissociation between object importance (relevance for the scene) and salience (relevance for attention). If an object obeys natural scene statistics, important objects are also salient. However, when natural scene statistics are violated, importance and salience are differentially affected. Object salience is modulated by the expectation about object properties (e.g., formed by context or gist), and importance by the violation of such expectations. In addition, the dependence of fixated locations within an object on the object's importance suggests an analogy to the effects of word frequency on landing positions in reading.

## Introduction

For the processing of natural scenes, object recognition and attention deployment are tightly intertwined. For example, preprocessing by an attention model aids computational object recognition (Rutishauser et al., 2004), an attention model can predict human recognition performance (Einhäuser et al., 2007) and biologically plausible object recognition models, such as HMAX (Riesenhuber and Poggio, 1999), can in turn serve as integral part when modeling categorical guidance of human attention towards complex objects (Zelinsky et al., under review). Since a shift of fixation is typically preceded by a re-allocation of attention towards the region to be fixated (Deubel and Schneider, 1996) and attention and eye-movement control share a common neural substrate (Rizzolatti et al., 1987), gaze orientation is a good proxy for the allocation of selective attention in a natural scene. Hence the relation between gaze and objects seems a key ingredient for the understanding of natural scene processing, with potential use for computational vision. Here we address how fixations on an object and the object's importance (Elazary and Itti, 2008; Spain and Perona, 2010) for the scene are modulated by a low-level feature: luminance contrast.

### Fixation probability and contrast

Gaze in natural scenes is to a large extent driven by high-level factors, such as the task (Buswell, 1935; Yarbus, 1967; Henderson et al., 2007; Einhäuser et al., 2008a) or spatial context (Torralba et al., 2006). Even in the absence of an explicitly instructed task (“free viewing”), high-level structures, such as faces (Cerf et al., 2008) or objects in general (Einhäuser et al., 2008b; Nuthmann and Henderson, 2010) seem to supersede low-level feature salience. Nonetheless, many models and experiments have addressed the interaction of gaze with low-level stimulus features. Most prominently, luminance contrast is long known to correlate with fixation probability in natural scenes (Reinagel and Zador, 1999; Krieger et al., 2000; Peters et al., 2005). Luminance contrast is also one of the key features of the saliency map model (Koch and Ullman, 1985), which is frequently used to predict fixations in natural scenes (Itti et al., 1998; Itti and Koch, 2000, 2001). However, the effect of luminance contrast is frequently confounded by its correlations with other features (Mannan et al., 1997; Baddeley and Tatler, 2006) or by a preference for high contrasts in an image center in combination with a preference to look straight ahead (“central bias,” Tatler, 2007). Furthermore, modifications of local contrast at random locations in foliage images have little effect for a broad range of contrasts: when contrast modifications become extreme, they attract attention, no matter whether contrast is increased or decreased (Einhäuser and König, 2003), which is incompatible with a causal effect of contrast on fixation probability. In line with object-based accounts (Einhäuser et al., 2008b; Nuthmann and Henderson, 2010) of fixations in natural scenes, such extreme modifications may be interpreted as being such an oddity relative to the background that they gain object or proto-object quality and thus attract attention. Such an interpretation is further supported by the fact that for large scale contrast modifications, the effect of contrast on salience (operationalized as the probability to fixate a certain contrast level) is linear (Einhäuser et al., 2006; Engmann et al., 2009). In a recent paper ('t Hart et al., 2013), we extended the contrast-modification paradigm to images that contain a single nameable object (rather than only foliage), and found the V-shape of contrast on fixation probability to persist when a random location is modified. However, when contrast modifications are applied to the object itself, the salience (probability to be fixated) of the object is increased for increased contrast and remains unaffected for decreased contrast. One possible reason for the latter null-effect was the absence of alternative objects to be looked at. Here we ask whether these results still hold, when competing objects are available in the scene and how the modifications of the object affect its probability to be regarded as important or “characteristic” of the scene. Thereby we assess the relation between salience (probability to be fixated) and importance (probability to be mentioned) of an object.

### Fixation distribution inside an object

Besides how frequently objects are fixated, a second important question on the relation between objects and eye movements pertains to the distribution of fixations relative to object boundary, given an object is fixated. Nuthmann and Henderson (2010) and Pajak and Nuthmann (2013) find that fixations have a bias towards the object's center, which depends on a variety of factors, such as objects size or the distance from the previous fixation. While our experiment is not explicitly designed toward this question, our data also allow us to explore whether the distribution of fixations within an object depends on how characteristic this object is perceived for the scene or on the contrast of the object.

## Methods

### Stimuli

As basis of our stimuli we used 93 photographs by the American photographic artist Stephen Shore from his “Uncommon Places” collection (Shore et al., 2004) that were provided in high-resolution by the artist and are available by request from Caltech's vision lab (http://vision.caltech.edu). Based on the data of Einhäuser et al. (2008b), we selected two objects in each stimulus, one that had frequently been named as characteristic (hereafter: “*common object*”) and one that had been named rarely (hereafter: “*rare object*”). The common object had to be named by at least twice the number of observers in the original study than the rare object, and both objects should be as closely matched as possible in size (number of pixels in the surface area). Of the 93 scenes, 72 were retained, in which a common and a rare object could be identified by these criteria. The factor with levels common and rare will be referred to as *frequency* throughout. It is important to note that the terms rare, common and frequency do not necessarily refer to an object property *per se*, but rather to the question at which frequency (rare or common) either object was named as scene-characteristic by the observers in the original 2008 experiment. The images were used at the same resolution (1024 × 768 pixels) as in Einhäuser et al. (2008b) and the surface area of objects was defined based on the object outlines created in the context of that study.

**Figure 1:**
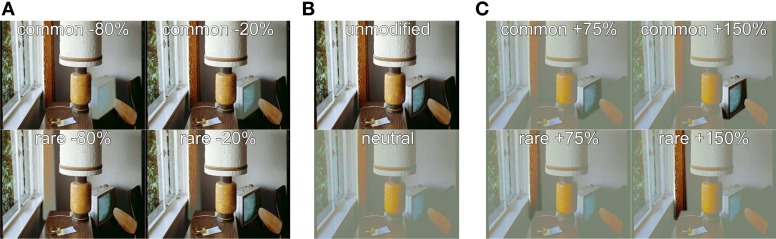
**Example stimuli. (A)** Stimuli from Experiment 1 with decreased luminance contrast. *Top row:* common object (TV set) modified, *bottom row:* rare object (curtain) modified, *left column:* −80% luminance contrast, *right column:* −20% luminance contrast. **(B)** *Top:* unmodified image [not used as stimulus in any experiment, but basis for modified stimuli of panel **(A)**], *bottom:* neutral stimulus [used in Experiment 2 and basis for the modified stimuli of panel **(C)**]. **(C)** Stimuli from Experiment 2 with increased luminance contrast. *Top row:* common object modified, *bottom row:* rare object modified, *left column:* +75% luminance contrast, *right column:* +150% luminance contrast. *Note that the sets of observers in experiment 1 and 2 were disjoint, and each basis image was only used once per observer, such that no observer could make any direct comparison between different modifications*.

Luminance contrast of one object within the scene was modified as follows:

$$\begin{array}{l} L_{\text{α}}(x,y)=\text{α}(L(x,y)-M)+M\;\;\;\text{if}(x,y)\text{ is within the object’s}\\ \;\;\;\;\;\;\;\;\;\;\;\;\;\;\;\;\;\;\;\;\;\;\;\;\;\;\;\;\;\;\;\;\;\;\;\;\;\;\;\;\;\;\;\;\;\;\;\;\;\;\;\;\;\;\;\text{surface area}\\ L_{\text{α}}(x,y)=L(x,y)\;\;\;\;\;\;\;\;\;\;\;\;\;\;\;\;\;\;\;\;\;\;\text{if}(x,y)\text{is outside the object’s}\\ \;\;\;\;\;\;\;\;\;\;\;\;\;\;\;\;\;\;\;\;\;\;\;\;\;\;\;\;\;\;\;\;\;\;\;\;\;\;\;\;\;\;\;\;\;\;\;\;\;\;\;\;\;\;\;\;\text{surface area} \end{array}$$

where *L*_α_ is the modified image, *L* is the neutral image (see below) and M half the of maximum of the luminance displayable on the screen (16.4 cd/m^2^). To avoid a sharp change of contrast at the boundary of the object, the pixels on and near the boundary of the object's surface area were blended between inside (modified) and outside (unmodified) contrast, using a Gaussian profile that was 5 pixels wide (sd) and centered on the boundary. In both experiments, two levels of modification α were used. In experiment 1, contrast was decreased with α= 0.2 and α = 0.8 for either the common or the rare object relative to the original image. These modifications will be referred to as −80% and −20% modification, respectively (Figure 1A). In Experiment 2, contrast was first globally decreased to 40% of the original value to allow all local increments to be displayable on the screen:

L(x,y)←0.4(L(x,y)−M)+M for all pixels

Relative to this neutral image (Figure 1B) contrast was locally increased as above, with α = 1.75 and α = 2.5. This implies a change of +75 or +150% relative to the neutral background (Figure 1C). The object with 150% modification matches then the object of the original image, and does therefore not exceed the screen's luminance range (0.33 cd/m^2^). The reduction of the global luminance contrast was necessary to allow the local modifications to span a large range in either direction. Since in a separate experiment with different stimulus material we verified that global modifications of luminance contrast do not alter relevant fixation behavior ('t Hart et al., 2013), we do not expect this to be a major restriction. However, we refrain from any direct comparison between the two experiments of the present study, as modifications are done relative to a different neutral stimulus (unmodified in Experiment 1, decreased to 40% of unmodified in Experiment 2). In Experiment 1, the neutral (unmodified) stimulus was not included, and only the two contrast modification levels were used. In Experiment 2, the neutral image was used in addition to the contrast modified images. This resulted in four conditions for Experiment 1 (two contrast levels times two frequency levels), and 5 conditions (contrast × frequency + neutral) for Experiment 2.

### Setup

Stimuli were displayed on a 19.7″ EIZO Flex Scan F77S CRT monitor (Samsung, Ridgefield Park, NJ, USA) run at 1152 × 864 px resolution at 100 Hz, stimuli (1024 × 768) were presented centrally within a grey frame of average stimulus luminance. Eye movements were monocularly recorded at 1000 Hz with an EyeLink 1000 infrared eye-tracking device (SR Research, Mississauga, ON, Canada). Observers were located ~42 cm from the screen, such that the image spanned about 33.7 × 25.3^°^ of visual angle. All presentation was performed with Matlab (MathWorks, Natick, MA, USA) using its psychophysics and eyelink toolbox extensions (Brainard, 1997; Pelli, 1997; Cornelissen et al., 2002).

### Participants

A total of 36 healthy adult volunteers participated in the experiments, 16 in Experiment 1, 20 in Experiment 2. All had normal or corrected-to-normal visual acuity, were fluent in German, and were naive to the purpose of the experiment. All experiments were conducted in accordance with the Declaration of Helsinki and procedures were approved by the responsible local committee (Ethikkomission FB04).

### Procedure

In both experiments, each stimulus was seen in each condition by four distinct observers, and no observer saw any scene more than once. Since there were four conditions in Experiment 1, the 16 observers had 18 trials per condition; with the five conditions of Experiment 2, the 20 observers had 14 trials per condition and the number of images was reduced from 72 to 70.

In each trial, observers first had to fixate centrally for at least 300 ms. Observers were then presented one stimulus for 3 s. After image offset, they were asked to rate how “artistic” the image was on a scale from 1 to 5, and to type “up to 5 keywords (concrete nouns)” that “described the scene well” thereafter. The 5 keywords will be referred to as “object mentions” throughout.

### Analysis

For all keywords provided by each observer it was checked whether they matched the common or rare object as defined above. This linking of object labels to objects was done manually, either by the authors (Experiment 1 and half of Experiment 2) or by paid annotators. None of the annotators had participated in the experiment. Periods of fixation were detected by the EyeLink software, using the manufacturers default thresholds for saccade detection (velocity larger than 35°/s and acceleration larger than 9.5°/s^2^) and defining fixations as periods between saccades. If a fixation was interrupted by a blink, the period before blink onset and after offset were counted as separate fixations.

For the analysis of fixation distributions inside an object, we adopted the bounding box scaling of Nuthmann and Henderson (2010). First, each object's bounding box is computed as the minimum rectangle that encompasses all the object's surface. If the object surface has disjoint sub-regions, two variants were possible. First, if a truly single object is disjoint by an occluder (e.g., a car behind a lamppost), it gets a single bounding box. Second, if a single object label refers to more than one object in the scene (e.g., two cars), the respective scene was discarded from this analysis. For the remaining 36 scenes, the bounding boxes then defined a coordinate system from the upper left corner at (−0.5, −0.5) to the bottom right corner at (+0.5, +0.5). Pixel coordinates within the bounding box were linearly mapped to this system, separately for *x* and *y* axes, resulting in a squared normalized bounding box, irrespective of the object's original aspect ratio. Since two observers of experiment 2 had no fixation on the rare object in the neutral condition for any of the scenes, they were excluded from this analysis.

All data was processed using Python 2.7.3 (http://www.python.org) with its numpy, scipy and pylab (Hunter, 2007) extensions; statistical analysis used R 2.14.1 (http://www.R-project.org; R Development Core Team, 2011).

## Results

### Neutral stimuli: confirmation of common/rare distinction

As first step, we verify that the distinction between common and rare objects, which was based on the data from Einhäuser et al. (2008b), also holds for the present data. Such replication is especially important, since the original experiment was performed in English, and the present experiment in German, and linguistic factors—such as word-frequency or length—may influence fixation behavior. In addition, the neutral images of Experiment 2 were reduced in global contrast relative to the unmodified stimuli used in the original paper. We find that rare objects were indeed named on average by less (1.29/4) observers than the common objects (2.74/4), and that this difference was significant [*t*_(69)_ = 7.00, *p* < 0.001, paired *t*-test with image as repeated measures]. Hence the use of the common/rare distinction is valid for the present experiment. Furthermore, rare and common object did not differ in luminance contrast (RMS contrast) for the neutral scene [*t*_(69)_ = 1.30, *p* = 0.199], which rules out unmodified contrast as potential confounding factor.

### Object properties

**Figure 2:**
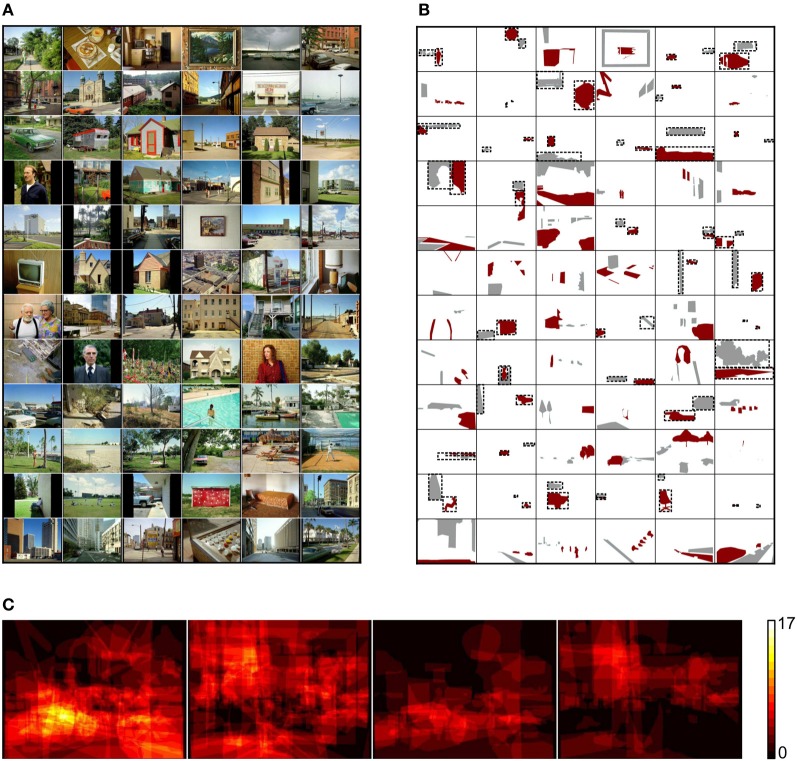
**Object properties. (A)** Seventy-two images from Stephen Shore's collection (Shore et al., 2004) used for the present experiments. Full resolution images are available for research upon request from Caltech's vision lab. Experiment 2 did not use the two final images in the lower right corner. **(B)** Surface area of common (gray) and rare (red) object for all images in **(A)**. For the 36 images for which bounding boxes were defined for both objects (see section Methods), the bounding boxes are given as dashed lines. **(C)** Object-maps (sum of all object surface areas); from left to right: 72 common objects, 72 rare objects, 36 common objects used for bounding box analysis, 36 rare objects used for bounding box analysis.

There is a potentially large set of features that could distinguish common objects from rare ones. Since spatial properties in the image plane could influence eye-movement patterns, we analyzed several of them for all images as well as separately for the images where we defined bounding boxes for both objects. We found no evidence for a difference between common and rare objects in any of the dimensions tested (Table 1), including size, different measures of eccentricity and aspect ratio. This does not exclude that other properties such as whether an object belongs to foreground or background or is perceived close or far contribute to an object being rare or common (Figures 2A,B for all objects and their surface area). Such properties, however, require already some degree of scene understanding, and are thus unlikely a trivial confound for the present analysis. However, common objects tend to be lower in the image than rare ones (Figure 2C). For all 72 images there is only a trend to this effect (at *p* = 0.06, Table 1); however, when restricting to the 36 images in which both objects have well-defined bounding boxes, the common objects have significantly lower positions than the rare objects (Table 1). Since eccentricity is indistinguishable (Table 1), this is, however, unlikely to confound fixation patterns within-scene or within-object in a trivial way, but may be related to a foreground/background distinction between common and rare objects, with regions lower in the image typically corresponding to foreground. Finally, since for objects touching the image boundary the measured center of gravity (relative to the visible portion) differs from the real center of gravity (relative to the actual object), we tested whether rare and common objects differ in this respect. We did not find a significant difference in the number of rare and common objects that touch the image boundary, neither in the full image set (43/72 and 31/72 respectively; χ^2^(1) = 0.229, *p* = 0.632) nor in the subset with well-defined bounding boxes (22/36 and 13/36; χ^2^(1) = 0.1564, *p* = 0.693). It should be noted, however, that all statements on object properties, as well as the property of being rare or common, are not meant to have any general implications for arbitrary scenes, keeping in mind that we deliberately selected a set of object-rich scenes, which are prone to photographer or artistic bias. The analysis of object properties is instead merely meant to ensure that the following results are not the consequence of a trivial difference between rare and common objects in the dataset used.

**Table 1:**
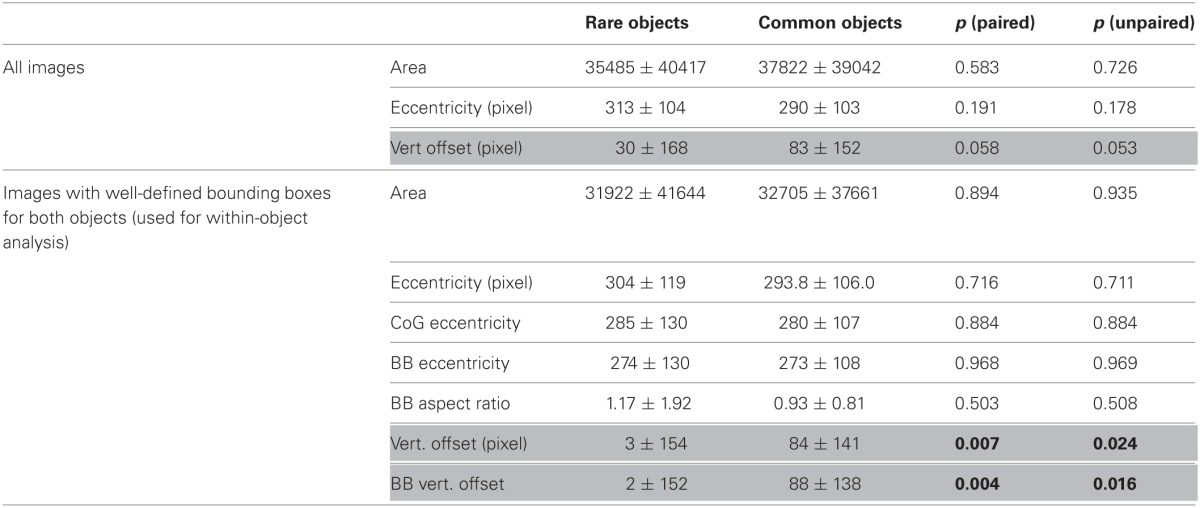
**Mean and standard deviation for spatial properties of rare and common objects**.

Statistical tests have been performed both with paired t-tests (i.e., using image as repeated measures) and unpaired. Paired tests assume that the competition between the two critical objects (rare/common) in the scene is relevant, while the unpaired tests follow the notion that distinct groups of observers were tested and there are many objects other than the critical one typically in the scene. For any measures, but vertical offset, we did not find any significant effects. Area: pixel count of object mask; eccentricity (pixel): average distance between any pixel of the object (or all its instances) from the image center; vertical offset (pixel): vertical component of this measure; CoG eccentricity: distance of the object's center of gravity to center of image; BB eccentricity: distance of the center of the object's bounding box to center of image; BB vertical offset: vertical component of this measure. All measures of vertical offset denote positions in pixels below the image midline (i.e., larger means lower). Note that, while the vertical offset of the center of gravity would be identical to the average vertical offset of all its pixels, this does not apply to the eccentricity (consider an annulus around the center for illustration, whose CoG's eccentricity is 0, while its average pixel eccentricity scales with the radius).

### Luminance contrast and object mentions

**Figure 3:**
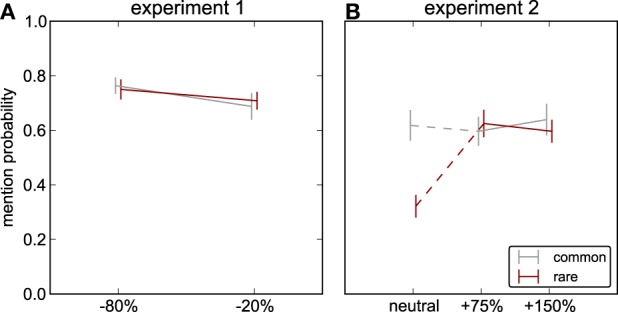
**Object mentions.** Probability that the modified object occurs in the list of keywords typed by observers to describe characteristic objects in the scene; *red*: rare objects, *gray*: common objects. **(A)** Experiment 1: decreases of luminance contrast. **(B)** Experiment 2: increases of luminance contrast and neutral image (no object modified, common and rare object as defined for the other conditions). Only the mention probability for the rare object in the neutral scenes in experiment 2 is significantly different from any other mention probability (details see Results).

First, we analyze whether the probability that an object is mentioned as characteristic for a scene (“object mentions”) depends on luminance contrast modifications. For both experiments we perform a two-factor ANOVA with factors frequency (common/rare) and contrast modification. For experiment 1 (contrast decrements), we see no main effect of frequency on object mentions [*F*_(1, 15)_ = 0.012, *p* = 0.92], and no effect of contrast [*F*_(1, 15)_ = 1.98, *p* = 0.18] and no interaction between the factors [*F*_(1, 15)_ = 0.40, *p* = 0.54] either (Figure 3A). In contrast, for Experiment 2 (contrast increments), we find a main effect of contrast [*F*_(2, 15)_ = 14.38, *p* < 0.001], of frequency [*F*_(1, 15)_ = 22.86, *p* < 0.001] and an interaction [*F*_(2, 15)_ = 12.58, *p* < 0.001, Figure 3B]. *Post-hoc* Tukey HSD tests show that this results from object mentions being significantly fewer for rare than for common objects in the neutral condition (see above), but for the contrast-modified conditions rare and common objects are indistinguishable (+75%: *p* = 0.999, +150%: *p* > 0.999). While for rare objects there is a significant difference between each of the modified and the neutral version (150%-neutral: *p* = 0.003, 75%-neutral: *p* < 0.001), this is not present for the common objects (150%-neutral: *p* > 0.999, 75%-neutral: *p* > 0.999) or between the two modification levels (rare: *p* = 0.999, common: *p* = 0.991). In sum, over both experiments, this shows that any of the tested modifications of contrast affects mention probability relative to neutral, while the strength of the modification is comparably irrelevant (thus no effect in Experiment 1, where neutral stimuli were not included). Data from Experiment 2 suggest that this effect primarily works through making rare objects more interesting, and thus indistinguishable from common objects, which already had raised interest—and thus mentions—because of their intrinsic/semantic importance for the scene.

### Object's luminance contrast and fixations

**Figure 4:**
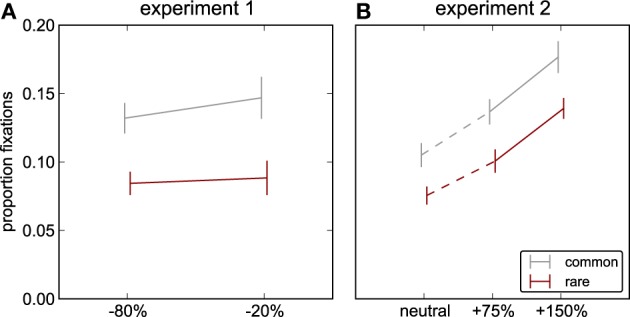
**Proportion fixations on the object.** The proportion of fixations landing on the modified object (*red*: rare, *gray*: common object modified). **(A)** Experiment 1: decreases of luminance contrast. **(B)** Experiment 2: increases of luminance contrast and neutral stimulus.

To test whether the proportion of fixations falling on an object increases with luminance contrast of the object, we perform a 2-factor ANOVA on the data of Experiment 1 with factors frequency (common/rare) and contrast (−80%, −20%) and observers as repeated measures. We find a main effect of frequency on fixation proportion [*F*_(1, 15)_ = 33.05, *p* < 0.001], but neither an effect of contrast [*F*_(1, 15)_ = 0.62, *p* = 0.44], nor an interaction [*F*_(1, 15)_ = 0.29, *p* = 0.60, Figure 4A]. In Experiment 2, we also find a main effect of frequency [*F*_(1, 38)_ = 27.6, *p* < 0.001], in addition to an effect of contrast [*F*_(2, 38)_ = 25.2, *p* < 0.001]. The relation between fixation proportion and contrast is monotonous and nearly linear (Figure 4B), and there is no interaction with the factor frequency [*F*_(2, 38)_ = 0.14, *p* = 0.87]. Taken together across Experiments 1 and 2, the frequency data confirm the original data on unmodified stimuli (Einhäuser et al., 2008b): common objects are fixated more frequently than rare objects. The contrast data are in line with the results on isolated objects described earlier ('t Hart et al., 2013): increasing contrast away from the natural “operating point” increases fixation proportion, while decreasing contrast has little effect despite the presence of other objects in the scenes.

### Fixation distribution within objects

**Figure 5:**
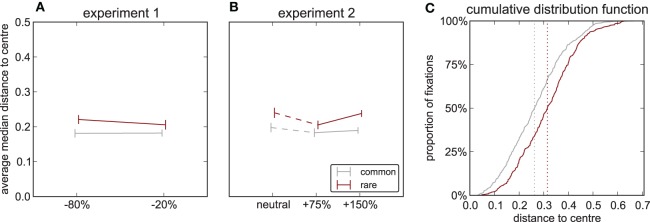
**Fixation distribution inside objects.** Median distance between position of fixations on the object and the center of its normalized bounding box, which by definition is a square of unit length. **(A)** Experiment 1. **(B)** Experiment 2. **(C)** Cumulative distribution function for rare (red) and common (gray) objects pooled over all experiments and contrast modifications. Vertical lines denote medians of distributions.

Analyses so far have concerned the fixations on an object irrespective as to where on the object the fixation landed; that is, fixations relative to the scene. Here we now address how fixations distribute *on* a fixated object; that is, fixations relative to the object's normalized bounding box. To quantify landing position in this object-centered coordinate system in a single number, we measure the distance to the bounding box center. Since the normalized bounding box of an object by definition is a square of unit length (see Methods), this distance ranges from ranges from 0 to $\sqrt{0.5}$. In both experiments we find a main effect of frequency [Experiment 1: *F*_(1, 15)_ = 7.37, *p* = 0.016, Experiment 2: *F*_(1, 17)_ = 13.6, *p* = 0.002], with the mean distance being larger for rare (exp 1: 0.302 ± 0.074, exp 2: 0.323 ± 0.074, mean ± sd) than for common objects (exp 1: 0.260 ± 0.046, exp 2: 0.278 ± 0.062). There is no effect of contrast [experiment 1: *F*_(1, 15)_ = 0.167, *p* = 0.688, experiment 2: *F*_(2, 34)_ = 1.115, *p* = 0.339] and no interaction [experiment 1: *F*_(1, 15)_ = 0.454, *p* = 0.511, Experiment 2: *F*_(2, 34)_ = 0.73, *p* = 0.489, Figures 5A,B]. Pooling the data over all experiments and contrast modifications shows two clearly separated distributions for rare and common objects (Figure 5C), further underlining that fixations on rare objects fall farther from the object's center. While we cannot exclude that an effect of contrast also does exist, which might be uncovered by experiments with larger samples and more statistical power, the effect of frequency even in this small sample is striking: the more important an object is for a scene, the more centrally it is fixated.

## Discussion

In the present study we relate fixations, the importance of an object for a scene as quantified by the number of observers mentioning the object, and a low-level stimulus feature, luminance contrast. We find that the importance of a rather unimportant (“rare”) object increases if its contrast is modified, though the strength of this modification seems to be comparably irrelevant. This suggests that the oddity rather than contrast *per se* may drive this effect. Second, we confirm that important objects are looked at more than unimportant ones. Third, objects with increased luminance contrast are looked at more, irrespective of whether or not they are important, while a decrease in an object's luminance contrast has nearly no effect. Fourth, important objects are fixated more centrally than unimportant objects.

For the present study we deliberately chose a set of stimuli of which most contain many objects and all were “composed” by the photographer according to his artistic conception. Hence, we did not aim at describing “natural” scene or object properties *per se*, but rather to study eye-movement properties with respect to objects, for which we tried to exclude trivial confounds (size, eccentricity) as far as possible. Since not all possible object properties can be captured, it is conceivable—and in fact likely—that some of the observed differences, in particular for the within-object fixation distributions, can be attributed to such hidden factors, which, for example, may relate to the interpretation of scene layout (foreground vs. background, near vs. far). Identifying such features that underlie an object's importance or salience is indeed a core question for contemporary computational vision (cf. Perona, 2010), and their dissociation therefore also of potential relevance for such applications.

Consistent with recent results on scenes with isolated objects, the effect of contrast modifications on fixations is restricted to positive modifications, while negative modifications show hardly any effect. Given the attractive effect of negative modifications at random locations (Einhäuser and König, 2003; Açik et al., 2009), it is well-conceivable that this absence results from a competition between two effects: a reduced visibility of the object yielding less fixations and an increased attractiveness, or importance, due to its deviation from expectations about scene statistics and context.

The data on neutral images confirm that when objects in natural scenes do not deviate from properties expected based on the statistics of the remainder of the scene, important objects draw more fixations than unimportant ones. This relative effect of importance on fixations is preserved, even when an originally unimportant object is made as “important” as the originally important object by changing its contrast, and even when the overall proportion of fixations on the objects increases through contrast increases. This suggests that not the importance of an object relative to the present scene is the dominant driver of fixations, but rather the expected importance of the object given global scene statistics. In other words, the relevance of an object for scene (“importance”) and its relevance for attention (“salience”) are dissociated.

Our measure of importance (frequency, common vs. rare) is related to a recently proposed definition by Spain and Perona (2010). Unlike these authors we, however, do not only estimate the probability of the object being mentioned *first*, but weigh all mentioned objects equally and count the number of observers mentioning them. This is mostly necessitated by our comparably small dataset (4 observers per neutral image) and the limited amount of items an observer is asked to name (maximally 5). However, given that the probability of a first mention and the number of observers mentioning an object are related for the unmodified images of the present set (Einhäuser et al., 2008b), this is unlikely to be a substantial constraint for the present purpose.

In a naming task similar to ours, Clarke et al. (2012) have recently described an interesting relation between the probability of an object being named in a scene and some linguistic factors, including lexical frequency. In turn, lexical frequency has a profound influence on temporal aspects of fixations in a scene, in particular for small objects (Wang et al., 2010). In this context it is interesting to observe that the “common” and “rare” objects were consistent between the Einhäuser et al. (2008b) study and the present one, which was conducted in a different language (English vs. German). The robustness across these experiments for neutral scenes, makes it seem unlikely that linguistic features, such as word-length of the label or its lexical frequency, confounded the present results. However, since language and cognition are often assumed to be in close interaction (linguistic relativity, Sapir-Whorf hypothesis), it cannot be excluded that conducting the study with native speakers of a different language family would change mentions and possibly eye-movement patterns.

The fact that common objects are fixated more centrally than rare objects may provide another interesting analogy: in reading, at a given word length, lower lexical frequency relates to a slight shift of the preferentially fixated location towards the beginning of the word (Nuthmann, 2006). Since in reading gaze enters a word usually from the word's beginning, this is analogous to the shift of fixated locations towards the periphery for rare objects, as observed here. Again, the effects on landing position inside the object seem to depend more on the original importance of the object (in the unmodified or neutral scene) than on its importance for the current stimulus, and therefore suggest an effect of scene statistics or context in guiding fixations even within objects. Recently several concepts and models were indeed successfully transferred from reading to natural scene viewing (Nuthmann and Henderson, 2010; Wang et al., 2010; Pajak and Nuthmann, 2013). However, the present notion of “rare” (i.e., rarely used to describe a particular image) does not map directly onto the linguistic notion, which refers to occurrence in large corpora. Hence “rare” objects (with respect to the specific scene) are by definition identified as less discriminative for a scene than common objects, while “rare” words (with respect to a corpus) might be more discriminative for a given text than common words. Whether “rare” objects are also rare with respect to large annotated databases (either in actual occurrence or in the annotation) is an open issue for future research. Nonetheless, exploring such analogies, and addressing the interaction of an object's features with its label's lexical properties in guiding gaze and attention, seems a promising path not only for eye-movement research in natural scenes, but for scene “understanding” in general.

### Conflict of interest statement

The authors declare that the research was conducted in the absence of any commercial or financial relationships that could be construed as a potential conflict of interest.
